# Interaction between Fiscal and Monetary Policy in a Dynamic Nonlinear Model

**DOI:** 10.1371/journal.pone.0118917

**Published:** 2015-03-23

**Authors:** Mario A. Bertella, Henio A. Rego, Celso Neris, Jonathas N. Silva, Boris Podobnik, H. Eugene Stanley

**Affiliations:** 1 Department of Economics, Sao Paulo State University (UNESP), Araraquara, SP, 14800–901, Brazil; 2 Center for Polymer Studies and Department of Physics, Boston University, Boston, MA, 02215, United States of America; 3 Federal Institute of Education, Science and Technology, São Luis, MA, 65076–091, Brazil; 4 Campinas State University (UNICAMP), Campinas, SP, 13083–857, Brazil; 5 Zagreb School of Economics and Management, Zagreb, 10000, Croatia; 6 Faculty of Civil Engineering, University of Rijeka, Rijeka, 51000, Croatia; 7 Faculty of Economics, University of Ljubljana, Ljubljana, 10000, Slovenia; University College London, UNITED KINGDOM

## Abstract

The objective of this study is to verify the dynamics between fiscal policy, measured by public debt, and monetary policy, measured by a reaction function of a central bank. Changes in monetary policies due to deviations from their targets always generate fiscal impacts. We examine two policy reaction functions: the first related to inflation targets and the second related to economic growth targets. We find that the condition for stable equilibrium is more restrictive in the first case than in the second. We then apply our simulation model to Brazil and United Kingdom and find that the equilibrium is unstable in the Brazilian case but stable in the UK case.

## Introduction

Inflation targeting (IT), i.e., adjusting interest rates to meet inflation goals, is a monetary policy strategy that has been adopted by a number of developed countries, including New Zealand (1990), Canada (1991), and the United Kingdom (1992), and also by several developing countries (Svensson [1]).

Mishkin [2] defines the IT regime as consisting of five elements: (i) a public announcement of a numerical inflation target (a point or a range) for a given time horizon, (ii) an institutional commitment to price stability as the ultimate goal of the monetary policy with other goals subordinate to it, (iii) the adoption of an information strategy that does not *solely* use variables such as monetary aggregates or the exchange rate as parameters to determine the policy instruments, (iv) a higher degree of transparency of the monetary policy strategy through communication with the public and the markets in relation to the plans, goals, and decisions of the monetary authorities, and (v) assigning the central bank greater responsibility in meeting inflation targets.

According to Bernanke et al. [3], the IT regime is the best monetary policy strategy because (i) it improves communication between the public and the monetary authorities and thus increases the agents’ capacity to forecast future inflation, and (ii) it disciplines the government’s monetary policy, thus giving it credibility. Credibility is the most important aspect of monetary policy as it avoids problems caused by time inconsistency (Barro and Gordon [4]; Kydland and Prescott [5]; Calvo [6]).

Barro and Gordon [4] use non-cooperative game theory to construct a “temptation” approach regarding policymakers and to compare the relationship between discretion and rules. They find that when there is a public announcement that an inflation target is going to be vigorously pursued, the agents’ (rational) expectations and their subsequent actions will contribute to the fulfillment of the target. If the monetary authority has high credibility, from the moment the announcement is made the agents will reduce their inflationary expectations, which will lead to a reduction in the cost of inflation. If the monetary policy deviates from the target in favor of discretionary conduct a so-called inflation bias is created. This discretionary conduct can be used to provide liquidity to the economy and, therefore, growth. Among the advocates of the effectiveness of the IT regime, Barro and Gordon [4] believe that discretion produces both transient effects and permanent inflationary ones. Kydland and Prescott [5], on the other hand, believe it produces only inflation and not transient effects. In other words, as the credibility of a central bank increases, its influence in reducing the cost of inflation also increases. Svensson and Woodford [7], Woodford [8,9], and Clarida *et al*. [10] divide the literature on credibility into two categories: (i) theoretical approaches that analyze the problem of persistent inflation when a monetary authority exercises discretionary behavior, and (ii) the study of monetary policies that take combating inflation seriously, understanding that if the economy experiences disinflation the social sacrifice may be greater than necessary.

The international literature that evaluates the experiences of countries that have implemented inflation targets is inconclusive regarding the effectiveness of this monetary policy strategy (for a survey of recent literature, see Svensson [1]). In a widely cited study, Ball and Sheridan [11] examined data from 20 countries, seven that adopted the IT regime before 1999 and 13 that did not. At the beginning of the IT regime in each country they recorded the first quarter in which the inflation target (or target interval) was pursued. Initial dates vary, with an interval of 1990:1 for New Zealand and 1995:1 for Spain, with a final analysis period in 2001 for all countries except Finland and Spain, due to the transition to the Euro. They compare these data for each country with data from time periods prior to the institution of the IT regime—a longer period beginning in 1960, a shorter period beginning in 1985, and the last full quarter period immediately prior to the institution of the IT regime.

Using macroeconomic indicators for the period sets between 1960 and 2001 for OECD member countries, the authors found no significant difference between the performance of countries that adopted IT and those that did not. They found that the average inflation rate and its volatility decreased considerably and that product growth exhibited greater stability in both countries that adopted IT and countries that did not. Mishkin and Posen [12] analyzed the cases of New Zealand, Canada, and the United Kingdom and found that the rate of inflation was reduced not because the regime was adopted but because disinflation was already taking place.

Neumann and Hagen [13] published a study with the same title as the study by Ball and Sheridan [11], but they concluded that IT does in fact reduce both the level of inflation and its volatility. Gonçalves and Salles [14] analyzed data from 36 emerging countries, 13 of which adopted IT some time between 1980 and 2005. They found that adopting the IT regime did have an effect on those 13 economies. The conclusions presented by Fraga *et al*. [15] agree with Gonçalves and Salles [14].

In this paper we use a dynamic model to examine the interaction between fiscal policy, as measured by public debt, and monetary policy as measured by a reaction function of a central bank. Changes in monetary policy due to deviations from their target always generate fiscal impacts. To our knowledge, the way in which this analysis was carried out has not yet been explored in literature. Thus, in the next section, we use a model that relates public debt to the search of an inflation target by means of a real interest rate. A regime of Growth Target (GT) rather than that of IT is suggested for countries that are in a recessionary environment. In the section that follows, we perform simulations for Brazil and United Kingdom in order to observe the trajectory of the public debt and real interest rate, based on the model above. The last section of the paper provides final considerations.

## Model

### 1. Inflation Targeting Regime

We first establish a simple model that relates fiscal policy by means of the public debt and monetary policy through interest rate. We define the change of the public debt in time as

$$\overset{\cdot }{B}=rB-(T-G)$$ (1)

where: $\overset{.}{B}=$change of the public debt in time;

*G* = public expenditure;

*T* = tax revenue;

*r* = real interest rate;

*B* = debt stock.

By denoting$b=\frac{B}{Y}$, i.e., the relation between public debt and product, (*Y*), we write:

$$\frac{\overset{\cdot }{B}}{Y}=rb-S$$ (2)

where$\frac{\left(T-G\right)}{Y}=S$, which corresponds to the public sector´s primary balance (deficit or surplus before expenditures with interest) as a share of GDP.

Deriving$b=\frac{B}{Y}$ in relation to time and considering$\overset{\cdot }{B}=rB-(T-G)$, we get:

$$\overset{\cdot }{b}=(r-g)b-S$$ (3)

where $g=\frac{\overset{\cdot }{Y}}{Y}$ corresponds to the growth rate of the economy.

On the other hand, considering a central bank focused only on optimal or desired inflation rate (*π* ^*^), that is, a central bank adopting inflation targets, then:

$$\overset{\cdot }{r}=\alpha (\pi -\pi^{*})$$ (4)

where *α* > 0, i.e., the change of the real interest rate in time varies according to the discrepancy between the effective rate of inflation (*π*) and the desired rate of inflation (π*). Thus, when the effective inflation is higher (lower) than expected, the real interest rate is raised (decreased) by the central bank. Here we are considering that a change in the public debt produces an alteration in the primary balance of public accounts in the same direction, *S*_*b*_ > 0, according to some empirical evidence, such as Bohn [16]. On the other hand, we will assume that the inflation rate is altered in the same direction when there is a change in the public debt as a share of GDP, i.e., *π*_*b*_ > 0.

Equations (3) and (4) form a system of differential equations, whose state variables are *b* and *r*. Partial derivatives given by the Jacobian matrix are:

$$\frac{\partial \overset{\cdot }{b}}{\partial b}=(r_{b}-g_{b})b+r-g-S_{b}$$ (5)

$$\frac{\partial \overset{\cdot }{b}}{\partial b}=(1-g_{r})b+(r-g)b_{r}-S_{r}$$ (6)

$$\frac{\partial \overset{\cdot }{r}}{\partial b}=\alpha \pi_{b}>0$$ (7)

$$\frac{\partial \overset{\cdot }{r}}{\partial r}=\alpha \pi_{r}<0$$ (8)

where we assume that *r*_*b*_ > 0, *g*_*b*_ < 0, *S*_*b*_ > 0, *g*_*r*_ < 0, *b*_*r*_ > 0, *S*_*r*_ > 0, *π*_*b*_ > 0 and *π*_*r*_ < 0.

The signs of equations (5) and (6) are inconclusive. Equation (7) is positive, indicating that an increase in public debt as a share of GDP raises inflation and, therefore, increases the change of the interest rate. On the other hand, Equation (8) is negative, showing that an increase in the interest rate reduces inflation and causes a reduction in the variation of the interest rate. Thus, the Jacobian matrix will be:

$$J=\left[\begin{array}{cc} \left(r_{b}-g_{b}\right)b+r-g-S_{b} & \left(1-g_{r}\right)b+\left(r-g\right)b_{r}-S_{r}\\ \alpha \pi_{b} & \alpha \pi_{r} \end{array}\right].$$

Therefore, the possible signs of the Jacobian matrix are:

$$J=\left[\begin{array}{cc} \pm & \pm \\ + & - \end{array}\right].$$

In order to simplify and reduce ambiguities, we will assume that *b*_*r*_ and *S*_*r*_ are null. Thus, the simplified Jacobian matrix is:

$$J=\left[\begin{array}{cc} \left(r_{b}-g_{b}\right)b+r-g-S_{b} & \left(1-g_{r}\right)b\\ \alpha \pi_{b} & \alpha \pi_{r} \end{array}\right].$$

Its corresponding signs are:

$$J=\left[\begin{array}{cc} \pm & +\\ + & - \end{array}\right].$$

Note that the stability condition for the point of equilibrium of a 2 x 2 dynamic system is Det *J* > 0 and Tr *J* < 0.

On dynamic analysis, we verify whether a position away from the equilibrium tends to converge or not, through the forces of the model, to an equilibrium point. Moreover, under this analysis, we learn about the specific character of the variable trajectory (e.g., node, saddle point and focus) toward (or away from) equilibrium. The stability analysis reveals the necessary and sufficient conditions for the variable trajectory to converge to the equilibrium point. For more on this topic see, e.g., Simon and Blume [17], Hoy et al. [18], and Shone [19].

Assuming that the difference between real interest rate and growth rate responses due to a public debt change is constant, i.e., *r*_*b*_-*g*_*b*_ = *c*, the determinant of *J* will be positive when Det

$$J=(cb+r-g-S_{b})\alpha \pi_{r}-(b-g_{r}b)\alpha \pi_{b}>0$$

or

$$(cb+r-g-S_{b})\alpha \pi_{r}>(b-g_{r}b)\alpha \pi_{b}$$ (9)

Note that *cb* > 0 can be understood as a change in the public debt stock due to a variation of the real interest rate and growth.

As (*b*-*g*_*r*_ *b*)*απ*_*b*_ > 0 and *απ*_*r*_ < 0, the required condition, even though still insufficient, is

$$cb+r-g-S_{b}<0$$ (10)

or

$$g+S_{b}>cb+r.$$

Moreover, the trace of matrix *J* should be negative, i.e.,

$$\left(cb+r-g-S_{b}\right)+\alpha \pi_{r}<0.$$ (11)

**Fig 1:**
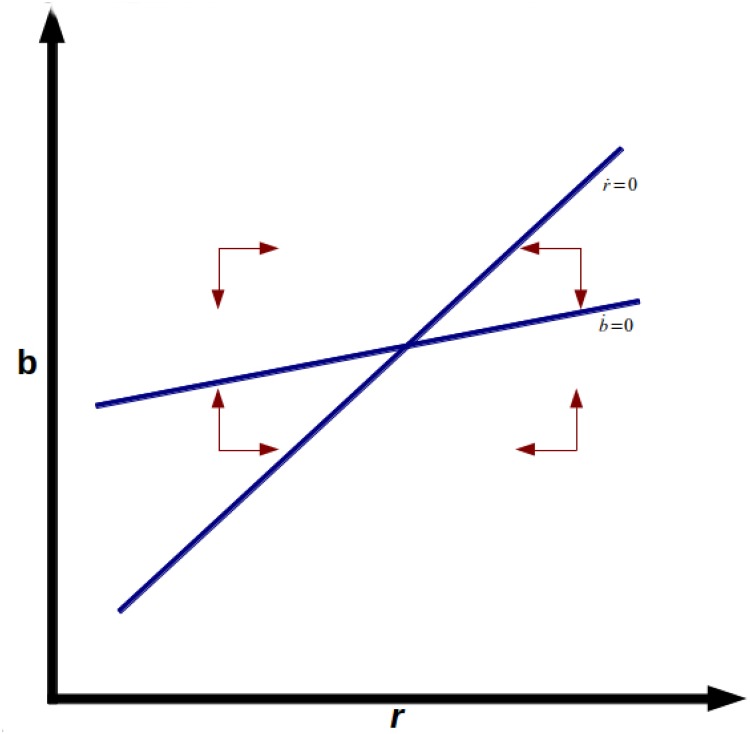
Stable Node.

**Fig 2:**
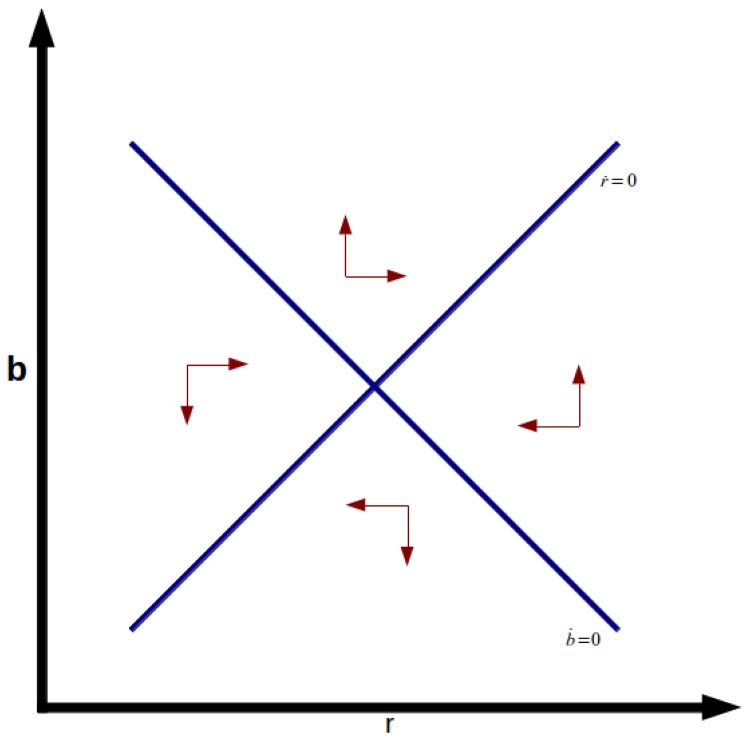
Saddle Point.

As *απ*_*r*_ < 0, then *cb*+*r*-*g*-*S*_*b*_ < 0 to ensure that the Tr*J* is negative. Note also that when Det *J* > 0, the Tr *J* < 0 necessarily, which excludes the possibility of having unstable node and focus. Here the growth rate of the economy plus the sensitivity of the public sector primary balance concerning the public debt should be higher than the sum of the real interest rate with a change of the public debt stock. Therefore when Det *J* > 0 (a required and sufficient condition) the signs of the trace and the determinant are attained, and the equilibrium is stable. Note also that, for a given *cb*, if the growth rate of the economy is so much lower than the real interest rate that there is no room for new reductions in the nominal interest rate—remembering that the government can accelerate the rate of inflation in order to reduce the rate of real interest—then the sensitivity of the primary balance of public accounts to the public debt *S*_*b*_ should increase sufficiently to keep public debt on a stable trajectory. The problem is that a decrease (or increase) in the public deficit (or surplus) can affect the rhythm of economic activity and further reduce the growth rate of the economy, and this can lead to a new round of public account adjustments so severe that the result is depression and social unrest. The solution is to encourage investment by making the business environment increasingly favorable to the private sector. Thus, when there is recession or depression, monetary policy should stimulate private sector investment by abandoning, at least temporarily, inflation targeting and focusing on economic growth targets with extremely low or even negative real interest rates. Fig. 1 shows the phase diagram of stable node equilibrium, and Fig. 2 shows the unstable saddle point equilibrium. Note also that changing the configuration of parameter signals can produce other kinds of equilibrium.

**Fig 3:**
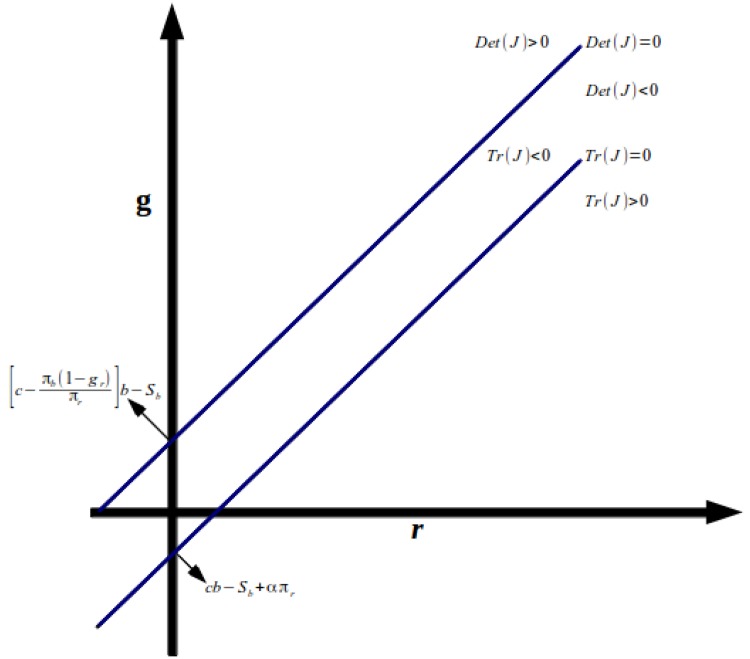
Stability and Instability of the Equilibrium on Plane *r*-*g*.

**Fig 4:**
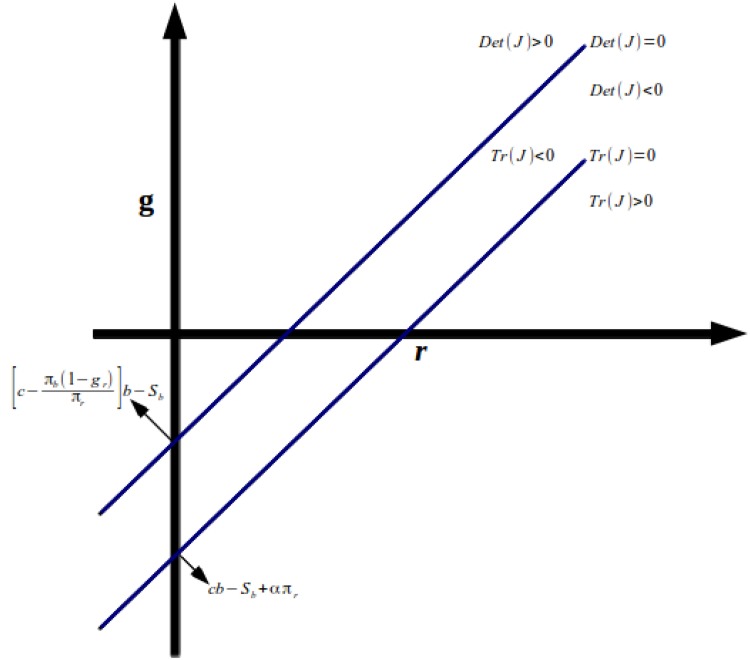
Stability and Instability of the Equilibrium on Plane *r*-*g*.

We also show Figs. 3 and 4 below, with the areas of stability and instability of the equilibrium on plane *r*-*g*. In Fig. 3, we consider$\left[c-\frac{\pi_{b}}{\pi_{r}}\left(1-g_{r}\right)\right]b>S_{b}$, while in Fig. 4 we have the opposite. As we can see, whether $\left[c-\frac{\pi_{b}}{\pi_{r}}\left(1-g_{r}\right)\right]b<S_{b}$ (Fig. 4), the possibilities to get a stable equilibrium are much higher than in Fig. 3.

Note further that the equilibrium value will occur when $\overset{.}{b}=0$ and$\overset{.}{r}=0$. In this case, the public debt as a share of GDP of equilibrium (b*) and the real interest rate of equilibrium (r*) correspond to:

$$b^{*}=\frac{S}{r-g}$$ (12)

$$r^{*}\in R$$

The real interest rate of equilibrium (r*) can be any value from the set of real numbers. It is sufficient that the effective inflation rate be equal to the expected inflation rate(*π* = π*). In order to ensure b* > 0, a primary public deficit is required, assuming that the growth rate of the economy is higher than the real interest rate. If it is lower, the public sector primary balance should be kept at a surplus.

### 2. Growth Targeting Regime

We next analyze an economy that is in an extreme recession or a depression. Here both differential equations for public debt and monetary policy are

$$\overset{\cdot }{b}=(r-g)b-S$$ (13)

$$\overset{\cdot }{r}=\beta (g-g^{*}),\beta >0.$$ (14)

Note that Equation (14) shows the interest rate as the result of the difference between the effective rate of growth (*g*) and the desired rate of growth (g*).

Assuming the same null derivatives as in the previous case, the Jacobian matrix will be

$$J=\left[\begin{array}{cc} cb+r-g-S_{b} & b-g_{r}b\\ \beta g_{b} & \beta g_{r} \end{array}\right].$$

When the relationship between public debt and economic growth is negative, i.e., *g*_*b*_ < 0, then the condition of a stable equilibrium will be

$$(cb+r-g-S_{b})\beta g_{r}>(b-g_{r}b)\beta g_{b}$$ (15)

and

$$(cb+r-g-S_{b})+\beta g_{r}<0$$ (16)

From expression (15), it is observed that, as the term (*b*-*g*_*r*_ *b*)*βg*_*b*_ < 0, it follows that the sufficient condition for the stability of the equilibrium is:

$$g+S_{b}>r+cb$$ (17)

which is the same condition as for Tr *J* < 0, because we assume that *g*_*r*_ < 0. If the real interest rate is null due to economic depression, then

$$g+S_{b}>cb$$ (18)

**Fig 5:**
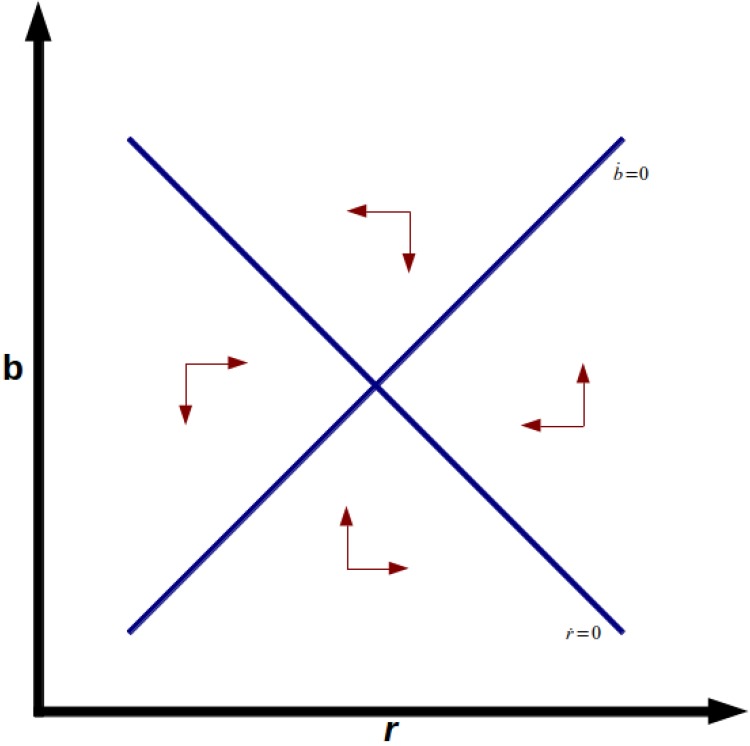
Stable Focus.

**Fig 6:**
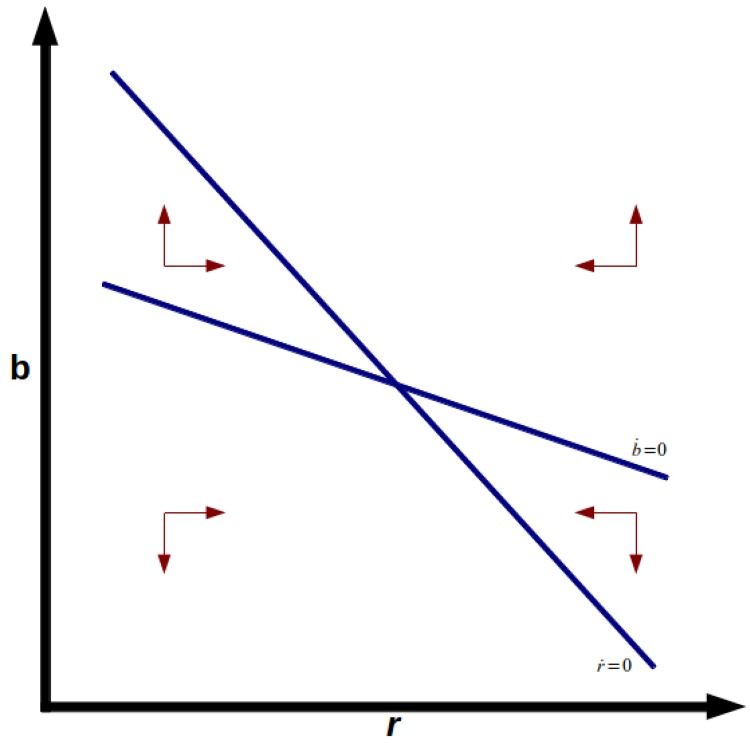
Saddle Point.

When the rate of economic growth plus the response of the primary surplus to a change in public debt is higher than *cb* it stabilizes the equilibrium. If the real interest rate is negative the growth rate can be negative, as long as condition (17) is respected. Note that when *g*_*b*_ < 0, the Det *J* is more likely to be positive than in the case described above—i.e., the determinant sign depends only on the result of *cb*+*r*-*g*-*S*_*b*_ and not on any additional condition. We thus conclude that an unstable equilibrium, e.g., such as saddle point equilibrium whose determinant of *J* is negative, is less likely to occur. Note also that when the Det *J* is positive the Tr *J* will be negative, and this excludes the possibility that an unstable node or focus equilibrium will occur. Note further that when there is a depression and the interest rate is targeting a specific level of economic growth, the real interest rate must be extremely low, null, or even negative which contributes substantially to lead public debt and growth rate into a stable equilibrium path. The problem is the length of time needed to attain this equilibrium. To shorten the time needed, the growth rate *g* must be raised by increasing exports and, if the fiscal situation permits, increasing government expenditures. Fig. 5 shows a phase diagram for the stable focus equilibrium, and Fig. 6 shows the unstable saddle point equilibrium. Other types of equilibrium can be found depending on the configuration of the parameter signals.

**Fig 7:**
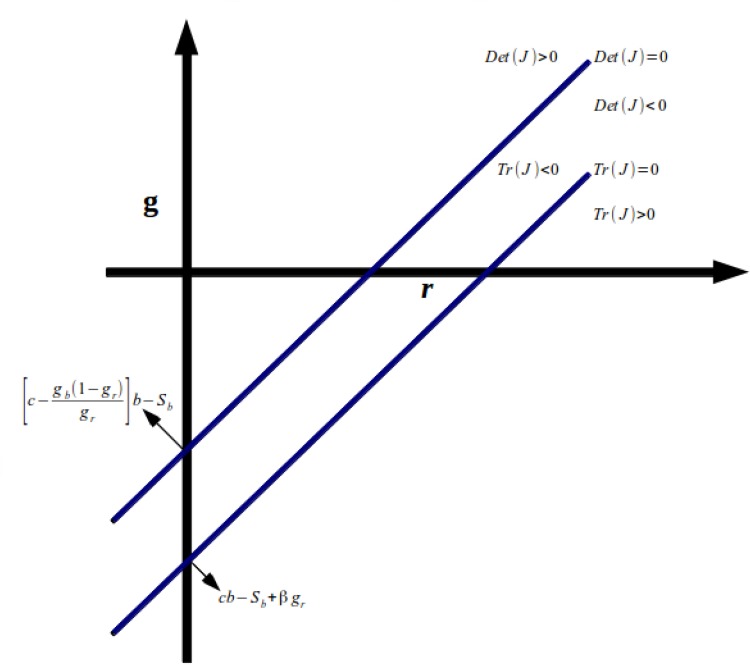
Stability and Instability Points on Plane *r*-*g*.

Assuming $S_{b}+(b-g_{r}b)\frac{g_{b}}{g_{r}}$>,*cb* we show in Fig. 7 the stability and instability points on plane *r*-*g*. Note also that as $-(b-g_{r}b)\frac{g_{b}}{g_{r}}<0$ and$-(b-g_{r}b)\frac{\pi_{b}}{\pi_{r}}>0$, the upright intercept of the Det *J* = 0 for the GT regime is smaller than the one for the IT regime. Note that a stable equilibrium is more probable under the GT regime than under the IT regime, as discussed above.

## The Brazilian and UK Cases

We now conduct simulations for the Brazilian and the UK cases based on the model defined above. We select these two countries because they adopted the inflation targeting regime, United Kingdom in 1992 and Brazil in 1999. They also allow us to contrast the economic behavior of a developed country with that of an emerging country. We find that their macroeconomic characteristics are very different. United Kingdom’s economy is characterized by moderate unemployment from December 2013 to February 2014 (6.9%), high public debt as a share of GDP (90.1%), low economic growth (1.8%), and a low inflation rate (2.6%). Brazil’s economy is characterized by low unemployment in December 2013 (4.6%), relatively low public debt (66.3%), low economic growth (2.3%), and a high inflation rate (6.2%). The data for United Kingdom are supplied by the Office for National Statistics [20], and the unemployment data for Brazil are supplied by the Brazilian Institute of Geography and Statistics [21]. All remaining data are supplied by the IMF [22–23]. In what follows we briefly describe their monetary and fiscal policies starting in 2000 and their respective simulations.

### 1. Brazilian Fiscal and Monetary Policy

During the 1999–2002 period, Brazil exercised a contractionary policy in order to maintain fiscal stability, and they implemented several important institutional mechanisms. The 1999–2001 three-year Action Plan established a growing trajectory for the primary surplus. This was preceeded by the 1998 Fiscal Stability Program, which put in place a fiscal restructuring that would meet targets set in collaboration with the International Monetary Fund (IMF). A floating exchange rate was adopted in January 1999. In 1999 the inflation targeting (IT) regime became the new anchor for guiding inflationary expectations. In 2000 the Fiscal Accountability Act (FAA) was instituted to control and manage public expenditures. During the 1999–2002 period, although the fiscal adjustment effort was strong and the primary surplus reached an average of 3.2% of GDP, the fiscal adjustment itself was unable to restrain the increase in public debt associated with the exchange rate and the contingent liabilities (Giambiagi [24]). In December 2002, government gross debt was 76.7% of GDP (Central Bank of Brazil [25]). The monetary policy of the 1999–2002 period was characterized by high nominal interest rates of about 19% per year, which led to an increase in expenditure to service the public debt. In this respect, although fiscal and monetary policies were successful in controlling inflation during this period, the average economic growth was 2.1% p.a.

When the leftist central government of President Lula da Silva took office in January 2003 they maintained the primary surplus policy initiated in the previous government of Fernando Cardoso for the first two years in order to control market insecurity. Throughout Lula da Silva’s first term in office the fiscal policy was kept contractionary, with an average primary surplus of 3.5% over the 2003–2006 period, and a gross average public debt for the period of 68.5% (the data on gross public debt are taken from the Central Bank of Brazil [25], and the Fiscal Monitor series by the IMF starting with 2006). The Fiscal Stability Program launched in 1999 was continued and the treasury and the central bank managed the public debt by reducing the base interest rate and exchange-rate-indexed bonds and by extending the medium term of the debt (Mendonça and Pinton, [26]). The monetary policy during Lula da Silva’s first term was also contractionary, with a base interest rate of about 18% per year, close to the average value of 19% of the previous government.

During Lula da Silva’s second term, which began in 2007, an inflexion occurred in the fiscal policy. Social expenditure, income transfer, and minimum salary were all increased, and a strategic component was adopted to attenuate future economic cycles. The response of the Brazilian anti-cyclical policy of fiscal stimulus to the 2008 crisis consisted of several actions, including expenditure increases, tax reductions, base interest rate reductions, and a gradual abandonment of the FAA (see, e.g., Neris Jr and Bertella, [27]). The primary surplus target was relaxed, fell to 2% in 2009, and stayed at an average level of 3% throughout Lula’s second term in office (2007–2010). Althought GDP growth in 2009 was –0.2%, it rose to 7.5% in 2010. The monetary policy became expansionary, the base interest rate reached the one-digit level during the 2009–2010 period, and it rose to an average level of 11.3% per year by the end of Lula’s government.

The next government of President Roussef was intended to be a continuation of President Lula’s second term and, in order to ensure the maintenance of growth in the subsequent periods, it extended the stimulus measures adopted earlier. The monetary policy was expansionary, with an average interest rate during the two initial years of 10.8% p.a. and with a tendency to drop. The fiscal policy was expansionary and the primary surplus target was reduced to an average of 2.7% for the two first years of her government (2011–2012). These factors, among others, strongly affected inflation. The target ceiling of 6.5% was reached by the end of 2011, which had not occurred since 2006, when inflation target was established at 4.5% with a band of + or—2 percentage points, and the growth of 7.5% for 2010 dropped to 2.7% in 2011. In 2012, inflation reached 5.8%, and GDP grew by 0.9%.

Table 1 shows the macroeconomic indicators for Brazil in accordance with IMF [22,23].

**Table 1:** Macroeconomic Data—Brazil (2013).

Public Debt (% GDP)	Real Growth (%)	Inflation (%)	Public Sector Primary Balance (% GDP)	Nominal Base Interest Rate (%) per year
66.3	2.3	6.2	1.9	10

Source: IMF [22,23].

To verify the dynamics of public debt and real interest rate, the data from table 1 were used. We also conducted several regressions in order to estimate the coefficients of the differential equation system presented in section 1 for the Brazilian case, including that of *S* = *f*(*b*) among others, to incorporate them into the equations for real interest rate and public debt. However, we found that the results were fruitless due to the limited quantity of available data, and due to the fact that the coefficients of the equations were statistically insignificant. The simulations for Brazil and the UK may have a limited character due to poor data set, conditioned in some way by the omitted variable bias. The omission of a variable causes a correlation between the error and the explanatory variables and therefore generates a bias and an inconsistency in the regression. However, we conducted the stability analysis for both countries based on what the data set could provide. Thus the differential equation system used for Brazil was

$$\overset{\cdot }{b}=(r-0.023)b-0.019$$ (19)

$$\overset{\cdot }{r}=0.5(0.1-r-0.045)$$ (20)

**Fig 8:**
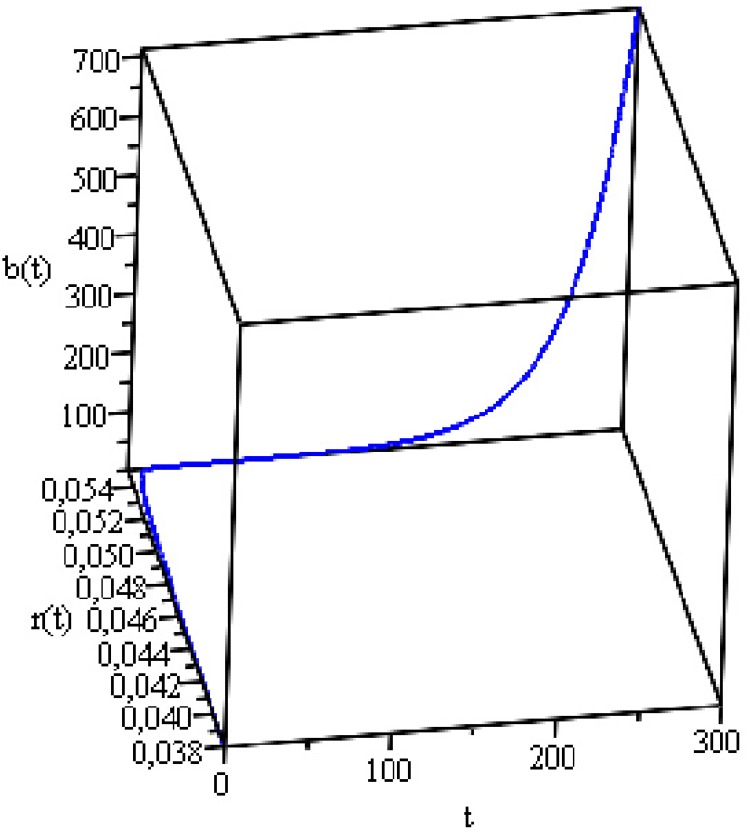
Public Debt and Real Interest Rate Dynamics.

Inflation corresponds to the difference between the nominal interest rate (10% per year) and the real interest rate, and the inflation target is 4.5% per year. Fig. 8 shows the dynamics of the two variables when the initial conditions are *b*(0) = 0.663 and *r*(0) = 0.038.

Fig. 8 shows that the joint trajectory of the interest rate and public debt is unstable. Although the point of equilibrium for the interest rate and public debt is *r* = 5.5% and *b* = 59.4% of GDP, respectively, the trajectory of the public debt exceeds this value due to the explosive behavior of the system (for the equilibrium point and a stability analysis for both countries, see S1 Appendix). We also used *α* = 0.5, as the various values found in the literature are inconclusive. Other values alter how quickly the monetary policy responds to discrepancies in inflation, but do not alter the quality of the analysis or its conclusion. Because the Brazilian economy is in full employment and restrictions are on the supply side and not on aggregate demand, it is not useful to discuss the economic growth regime via real interest rate—the approach we will use when we analyze the UK economy.

### 2. The UK Fiscal and Monetary Policy

Between the late 1990s and 2007, the UK fiscal policy was concerned with keeping the public budget relatively balanced throughout the economic cycle with a public deficit of about 2% of GDP, and a public debt close to 40% of GDP (Sawyer [28]).

When the financial crisis erupted in 2008, the UK government, still in the hands of the Labor Party, adopted a more expansionary fiscal policy: they reduced the value added tax from 17.5% to 15% (but increased it back by the end of 2009), and increased public expenditure by 3 billion pounds. Thus for the fiscal year 2008 the public deficit rose to 5% of GDP, and for 2009 to 11.3% of GDP [29]. Prior to 2007 public debt was approximately 40% of GDP. It jumped to 52% of GDP in 2008 and to 67% in 2009 [29]. The new UK conservative government, which took office in May 2010, changed the fiscal policy. It responded to growing public deficit and debt by rapidly cutting expenditures (by 5 billion pounds) and raising taxes (by 2.8 billion pounds). According to HM Treasury [30], the goal was to eliminate the structural public deficit by 2014–2015 and attain a surplus of 0.8% of GDP by 2015–2016.

The inflation targeting regime in the United Kingdom was established in 1992 when the target was set at 2% per year (Bank of England [31]). The inflation rate, which was at 7.5% per year in 1991 (World Bank [32]), dropped to 2% in 1993, and remained at approximately this level until 1998 when the UK central bank became officially independent of the political authority. From 1998 to 2004 inflation remained between 1% and 2% per year but then increased until it reached its highest level in 2011, i.e., 4.5% p.a. (World Bank [32]). Note that the financial crisis of 2008 caused the monetary policy to be expansionary as well, with a reduction in the base interest rate from 5% per year in September 2008 to 0.5% per year in March 2009, the level at which it has remained to date (April, 2014). Like the US Fed, the UK central bank has also committed itself to purchasing public and private securities (*quantitative easing*) in order to expand the liquidity of the economic system (Bank of England [31]).

Although there is currently an expansionary monetary policy in the United Kingdom, the rate of growth and recovery of economic activity may be put in jeopardy by the restrictive fiscal policy practiced by the conservative government of Prime Minister James Cameron. Table 2 contains the macroeconomic indicators according to the IMF [22, 23].

**Table 2:** Macroeconomic Data–United Kingdom (2013).

Public Debt (% GDP)	Real Growth (%)	Inflation (%)	Public Sector Primary Balance (% GDP)	Nominal Base Interest Rate (%)
90.1	1.8	2.6	-4.5	0.5

Source: IMF [22,23].

We used the data from Table 2 to verify the dynamics of public debt and real interest rate indicated by the system of differential equations shown in sec. 1. We also conducted several regressions in order to estimate the coefficients of the differential equation system presented in sec. 1 for the UK case, including that of *S* = *f*(*b*) among others, to incorporate them into the equations for real interest rate and public debt. However we found that the results were fruitless due to the limited quantity of available data and due to the fact that the coefficients of the equations were statistically insignificant. So the system of differential equation becomes

$$\overset{\cdot }{b}=(r-0.018)b+0.045$$ (21)

$$\overset{\cdot }{r}=0.5(0.005-r-0.02).$$ (22)

**Fig 9:**
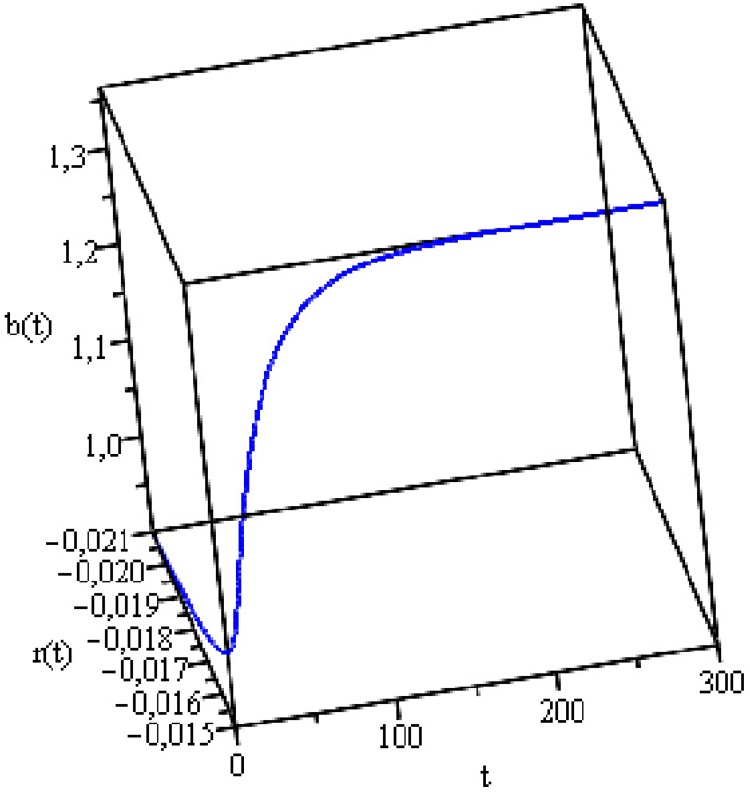
Public Debt and Real Interest Rate Dynamics.

Here note that inflation corresponds to the difference between the nominal interest rate (0.5% per year) and the real interest rate, and that the inflation target is 2% per year. Fig. 9 shows the dynamics of the two variables when the initial conditions are *b*(0) = 0.901 and *r*(0) = -0.021.

**Fig 10:**
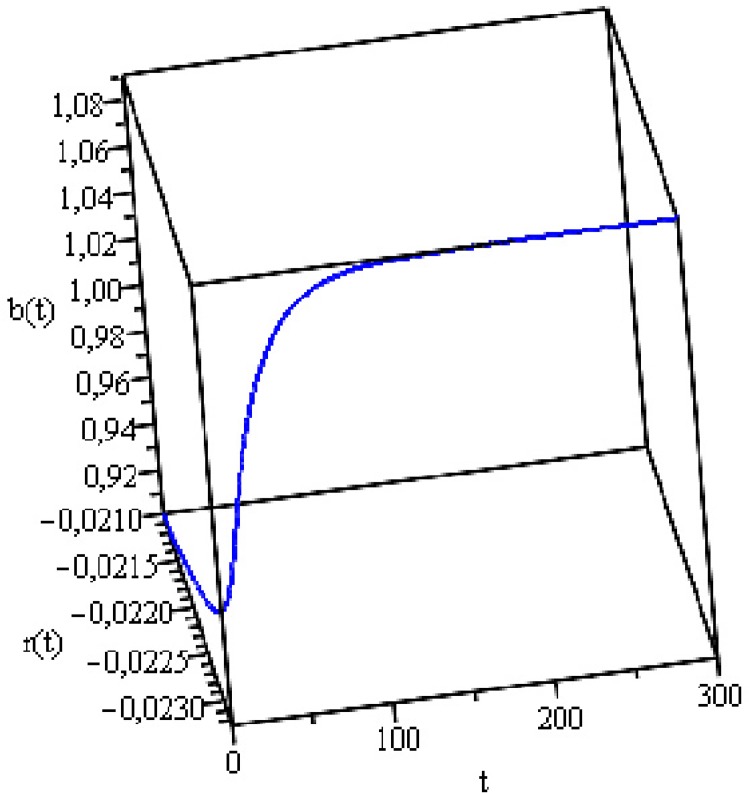
Public Debt and Real Interest Rate Dynamics. (Growth Targeting Regime).

Fig. 9 shows that the UK public debt is stable. It requires approximately 150 time units to reach its equilibrium with 136.4% of GDP and a real interest rate of -1.5% per year. The value α = 0.5 was used for the same reasons as in the Brazilian case. On the other hand, Fig. 10 shows that if economic growth targets are adopted the stable equilibrium is reached with a real interest rate of -2.3% per year and a public debt of 109.8% of GDP. This simulation retained all data from the previous case (see Fig. 9) and established the growth target at 2% per year. Because the relationship between economic growth and real interest rate is negative, we set *g* = -0.86*r*. We used the value 0.86 because it is compatible with the 2013 growth rate of 1.8%. Note that the public debt values and the real interest rate of equilibrium are lower than those in the previous case (see Fig. 9), as well as the time required to reach this equilibrium (approximately 100 time units). Note also that the UK central bank practices a monetary policy that prioritizes real variables, e.g., the growth rate, to the detriment of the inflation target. The base interest rate is low (0.5% per year) and the inflation rate well above the target (2% per year) since 2010 (see table 3).

**Table 3:** Annual Inflation Rate (%)–United Kingdom.

2010	2011	2012	2013
3.3	4.5	2.8	2.6

Source: World Bank [32].

## Final Considerations

In this study we have investigated the dynamic interaction between monetary policies that attempt to meet inflation or growth targets by setting an interest rate rule and fiscal policies, measured by the public debt. We believe this approach has not been taken previously and that is has not appeared in the literature.

We first verified that when the growth rate of the economy is higher than the difference between *cb* [see Equation (9)] and the real interest rate, and the primary balance of public accounts responds to public debt, the behavior of the public debt and the real interest rate will be non-explosive. This condition is required but not sufficient for an IT regime. On the other hand, under a GT regime this condition is sufficient to provide stable equilibrium. We have assumed that when a government keeps the relationship between public debt and the primary balance of public accounts positive, its actions are serious and responsible. Empirical evidence supports this assumption.

We next simulated a theoretical model of the economies of Brazil and the UK. We observed that the path of public debt is explosive in Brazil and stable in the UK. Although governments can take measures to change the trajectory, e.g., by reducing real interest rates to stimulate economic growth, this particular measure is appropriate only when the economy is far from full employment. This policy was tested in Brazil between August 2011 and October 2012 when the central bank voluntarily reduced the base interest rate, but the only result was accelerated inflation. Thus any measure taken by Brazil should be fiscal—increasing the primary surplus—and the central bank should concentrate on combating inflation and regaining credibility. In the UK case the central bank pursued a monetary policy that focused on a real variable, e.g., the growth rate, at the expense of an inflation target. The UK government has made important fiscal adjustments in order to balance the budget for future years, but these ajustments have negatively affected economic growth and employment levels—an outcome neither desired by the government nor within a time acceptable to UK society.

This work has two limitations. The first is its orthodox character. Prior to the financial crisis of 2008 several authors criticized the direction of conventional economic theory and presented alternative macroeconomic theories (e.g., Davidson [36] and Minsky´s financial instability hypothesis [37–39]). After the crisis dissatisfaction with mainstream theory grew, as many authors proposed a “new macroeconomic consensus” in which monetary policy and inflation targeting took center stage. On the one hand there was post-Keynesian criticism that the regime of inflation targeting would not deliver low inflation and that the primary objective of monetary policy should be financial stability rather than inflation (Arestis and Sawyer [33]). There were a variety of similar approaches, including those of Allington et al. [40], Bezemer [41], and Werner [42]. On the other hand complexity theorists (Colander et al. [34], Gatti et al. [35]) proposed that emerging aggregate results were not a simple sum of the behavior of individual agents—which is the assumption of conventional macroeconomic theory. In addition, there was explicite recognition by a number of the leading exponents of the dominant economic theory that there were flaws in their approach (Blanchard et al. [43]). Thus in our paper we also have investigated the dynamics between fiscal and monetary policy using a less orthodox interest rate rule in which the central bank pursues a real variable (growth rate), although we acknowledge that this variable may not be the most appropriate and its determination requires further research. The second limitation of our paper is the simplified nature of our theoretical model. We have used variables that seek to answer one simple question: what is the nature of the dynamic between fiscal policy and monetary policy and what conditions are sufficient for them to achieve stabililty? To enrich this analysis, private debt must be incorporated. A possible source of inspiration for this could be the seminal works by Minsky [38–40] and their corresponding formal models (e.g., Delli Gatti et al. [44] and Keen, [45]), or more conventional work, e.g., that done by Bernanke and Gertler [46], Kiyotaki and Moore [47], and Eggertsson and Krugman [48].

For this work we used a deterministic nonlinear dynamical system since it does not include any stochastic term. This does not mean that the behavior of the system is predictable. Nonlinear deterministic systems can produce chaotic behavior. In chaotic systems the uncertainty of a model forecast increases exponentially with elapsed time and a meaningful prediction cannot be made over a time interval that is approximately two to three times larger than the Lyapunov time. For the case in which meaningful predictions cannot be made, the chaotic system seems to be random. A further development of this work will include stochastic terms in model equations.

## Supporting Information

S1 Appendix Equilibrium point and stability analysis (Brazil and United Kingdom). (DOCX)

## References

[1] Svensson L (2010) “Inflation Targeting”. *NBER Working Paper Series*, n. 16654.

[2] Mishkin F (2000) “Inflation Target in Emergent Market Countries”. *NBER Working Paper Series*, n. 7618.

[3] Bernanke B, Laubach T, Mishkin F, Posen A (1999) *Inflation Targeting: Lessons from the International Experience*. Princeton University Press.

[4] Barro R, Gordon D (1983) “Rules, Discretion and Reputation in a Model of Monetary Policy”. *Journal of Monetary Economics*, 12 (July): 101–121.

[5] Kydland F, Prescott E (1977) “Rules rather than Discretion: The Inconsistency of Optimal Plans”. *Journal of Political Economy*, 85 (June); 473–492.

[6] Calvo G (1978) “On the Time Consistency of Optimal Policy in the Monetary Economy”. *Econometrica*, v. 46, n. 4, p. 1411–1428.

[7] Svensson L, Woodford M (2003) *Implementing Optimal Policy through Inflation-Forecast Targeting*. NBER Working Paper, n. 9747.

[8] Woodford M (1999a) “Commentary: How Should Monetary Policy Be Conducted in an Era of Price Stability” In: *New challenges for Monetary Policy*, Federal Reserve Bank of Kansas City, p. 277–316.

[9] Woodford M (1999b) “Optimal Monetary Policy Inertia”. *NBER Working Paper Series*, n. 7261.

[10] Clarida R, Gali J, Gertler M (1999) “The Science of Monetary Policy: a New Keynesian Perspective”. *NBER Working Paper Series*, Cambridge, MA, 7147, May.

[11] Ball L, Sheridan N (2003) “Does Inflation Targeting Matter?” *NBER Working Paper Series*, n. 9577, p. 1–47.

[12] Mishkin F, Posen A (1997) “Inflation Targeting: Lessons from Four Countries”. *Economic Policy Review*, Federal Reserve Bank of New York, August.

[13] Neumann M, von Hagen J (2002) “Does Inflation Target Matter?” *Economic Review of the* Federal *Reserve Bank of St*. *Louis*, 84, 127–148.

[14] Gonçalves C, Salles J (2008) “Inflation Targeting in Emerging Economies: What do the Data Say?”, *Journal of Development Economics*, 85: 312–318.

[15] Fraga A, Goldfajn I, Minella A (2003) “Inflation Targeting in Emerging Market Economies”. *NBER Working Paper Series*, n. 10019.

[16] Bohn H (1998) “The Behavior of US Public Debt and Deficits”, *The Quarterly Journal of Economics*, 113 (3).

[17] Simon C, Blume L (1994) *Mathematics for Economists*, W. W. Norton & Company.

[18] Hoy M, Livernois J, McKenna C, Rees R, Stengos T (2001) *Mathematics for Economics*, MIT Press, 2nd ed.

[19] Shone R (2002) *Economic Dynamics*: *Phase Diagrams and their Economic Application*, Cambridge University Press, 2nd ed.

[20] Office for National Statistics (2014) *Statistical Bulletin*: *Labour Market Statistics*, April, at http://www.ons.gov.uk/ons/rel/lms/labour-market-statistics/april-2014/statistical-bulletin.html.

[21] Brazilian Institute of Geography and Statistics (2014) *Aggregate Database (SIDRA)* at http://www.sidra.ibge.gov.br/bda/pesquisas/pme/default.asp?o=20&i=P.

[22] IMF (2014a) *World Economic Outlook*: *Recovery Strengthens*, *Remains Uneven*, April, at http://www.imf.org/external/pubs/ft/weo/2014/01/pdf/text.pdf.

[23] IMF (2014b) *Fiscal Monitor*: *Public Expenditure Reform—Making Difficult Choices*, April, at http://www.imf.org/external/pubs/ft/fm/2014/01/pdf/fm1401.pdf.

[24] Giambiagi F (2007) “Seventeen Years of Fiscal Policy in Brazil: 1991–2007”. A Text for Discussion no. 1309. (Dezessete Anos de Política Fiscal no Brasil: 1991–2007. Texto para Discussão nº 1309) IPEA—Rio de Janeiro. November.

[25] Central Bank of Brazil (2013) *Gross and Net General Government Debt Historical Series (Methodology Effective as of 2007)*, at http://www.bcb.gov.br/?NPDDEBT.

[26] Mendonça H, Pinton O (2012) “Behavior of the Brazilian Fiscal Policy in the 21st Century: An Analysis of Fiscal Impulse”. (O Comportamento da Política Fiscal Brasileira no século XXI: Uma Análise a partir do Impulso Fiscal) *Revista Economia*, v.13, n.2, p. 281–301, May-Aug.

[27] Neris C Jr, Bertella MA (2013) “From Financial to Sovereign Debt Crisis: A Debate on Fiscal Policy”. (Da Crise Financeira à Crise da Dívida Soberana: Um Debate sobre a Política Fiscal) *Análise Econômica*, Porto Alegre, year 31, n. 59, p. 123–143, 3.

[28] Sawyer M (2011) “UK Fiscal Policy After the Global Financial Crisis”, *Contributions to Political Economy* 30, 13–29.

[29] IMF (2013) *Fiscal Monitor*: *Taxing Times*, October, at http://www.imf.org/external/pubs/ft/fm/2013/02/pdf/fm1302.pdf.

[30] HM Treasury. (2010) *Budget 2010*: *Securing the Recovery* London: The Stationery Office, HC451, at http://webarchive.nationalarchives.gov.uk/20100413203226/http:/www.hm-treasury.gov.uk/d/budget2010_complete.pdf.

[31] Bank of England (2013) *Monetary Policy Trade-offs and Forward Guidance*, August, at http://www.bankofengland.co.uk/publications/Documents/inflationreport/2013/ir13augforwardguidance.pdf.

[32] World Bank (2013) *Inflation*, *Consumer Prices (annual%)*, at http://data.worldbank.org/indicator/FP.CPI.TOTL.ZG.

[33] Arestis P, Sawyer M (2010) “What Monetary Policy after the Crisis?”, *Review of Political Economy*, 22:4, 499–515.

[34] Colander D, Haas A, Goldberg M, Juselius K, Kirman A, et al (2009) “The Financial Crisis and the Systemic Failure of Academic Research”, *Crit Rev* 21:249–267.

[35] Gatti D D, Gaffeo E, Gallegati M (2010) “Complex Agent-based Macroeconomics: A Manifesto for a New Paradigm”, *Journal of Economic Interaction and Coordination*, 5:111–135.

[36] Davidson P (1994) *Post Keynesian Macroeconomic Theory*: *A Foundation For Successful Economic Policies For The Twenty-First Century*, Cheltenham Elgar.

[37] Minsky H (1975) *John Maynard Keynes*. New York: Columbia University Press.

[38] Minsky H (1982) *Can ‘It’ Happen Again*? *Essays on Instability and Finance*, M.E. Sharpe, Armonk, NY.

[39] Minsky H (1986) *Stabilizing an Unstable Economy* Twentieth Century Fund Report, Yale University Press, New Haven and London.

[40] Allington N F B, McCombie J S L, Pike M (2011) "The Failure of the New Macroeconomic Consensus: From Non-ergodicity to the Efficient Markets Hypothesis and Back Again", *International Journal of Public Policy* 7(1–3): 4–21.

[41] Bezemer D J (2011) "The Credit Crisis and Recession as a Paradigm Test", *Journal of Economic Issues* 45: 1–18.

[42] Werner R (2012) "Towards a New Research Programme on ‘Banking and the Economy’—Implications of the Quantity Theory of Credit for the Prevention and Resolution of Banking and Debt Crises." *International Review of Financial Analysis*, vol. 25, 1–17.

[43] Blanchard O, Dell'Ariccia G, Mauro P (2010) "Rethinking Macroeconomic Policy", *Journal of Money*, *Credit*, *and Banking* 42: 199–215.

[44] Delli Gatti D, Gallegati M, Gardini L (1994) “Complex Dynamics in a Simple Macroeconomic Model with Financing Constraints”, in Dymski G. and Pollin R. (eds.), *New Perspectives in Monetary Macroeconomics*: *Explorations in the Tradition of Hyman Minsky*, Ann Arbor: The University of Michigan Press, 51–76.

[45] Keen S (1995) “Finance and Economic Breakdown: Modeling Minsky’s Financial Instability Hypothesis”, *Journal of Post Keynesian Economics*, v. 17, n. 4, p. 607–35.

[46] Bernanke B, Gertler M (1989) “Agency Costs, Net Worth and Business Fluctuations”, *American Economic Review*, 79, 14–31.

[47] Kiyotaki N, Moore J (1997) “Credit Cycles”, *Journal of Political Economy*, 105, 211–248.

[48] Eggertsson GB, Krugman P (2012) "Debt, Deleveraging, and the Liquidity Trap: A Fisher-Minsky-Koo Approach", *Quarterly Journal of Economics* 127: 1469–1513.

