# Supplementary material for: Interaction between Fiscal and Monetary Policy in a Dynamic Nonlinear Model
Source: PLoS One. 2015 Mar 23;10(3):e0118917. doi: 10.1371/journal.pone.0118917 (PMC4371610; doi:10.1371/journal.pone.0118917)
Supplement: S1 Appendix — (DOCX) [file pone.0118917.s001.docx]

**Appendix**

**Brazil**


.

1. Equilibrium Point


.

2. Stability Analysis

$J=\left[\begin{matrix} r-0.023 & b \\ 0 & -0.5 \end{matrix}\right]$.

The Jacobian matrix at the equilibrium point is:

$$J=\left[\begin{matrix} 0.055-0.023 & 0.594 \\ 0 & -0.5 \end{matrix}\right]$$

Det *J* = -0.016 (Saddle Point) (Unstable Equilibrium Point).

**United Kingdom (Inflation Target)**


.

1. Equilibrium Point


.

2. Stability Analysis

$J=\left[\begin{matrix} r-0.018 & b \\ 0 & -0.5 \end{matrix}\right]$.

The Jacobian matrix at the equilibrium point is:

$J=\left[\begin{matrix} -0.015-0.018 & 1.364 \\ 0 & -0.5 \end{matrix}\right]$.

Det *J* = 0.0165 > 0 and Tr *J* = -0.533 $\to$ Stable Equilibrium Point.

**United Kingdom (Growth Target)**


.

1. Equilibrium Point


.

2. Stability Analysis

$J=\left[\begin{matrix} r-0.018 & b \\ 0 & -0.43 \end{matrix}\right]$.

The Jacobian matrix at the equilibrium point is:

$J=\left[\begin{matrix} -0.023-0.018 & 1.098 \\ 0 & -0.43 \end{matrix}\right]$.

Det *J* = 0.01763 > 0 and Tr *J* = -0.471 $\to$ Stable Equilibrium Point.
